# Identification of a Novel Actin-Binding Domain within the Rho Guanine Nucleotide Exchange Factor TEM4

**DOI:** 10.1371/journal.pone.0041876

**Published:** 2012-07-24

**Authors:** Natalia Mitin, Kent L. Rossman, Channing J. Der

**Affiliations:** Lineberger Comprehensive Cancer Center and Department of Pharmacology, University of North Carolina at Chapel Hill, Chapel Hill, North Carolina, United States of America; BioScience Project, United States of America

## Abstract

Spatio-temporal activation of Rho GTPases is essential for their function in a variety of biological processes and is achieved in part by regulating the localization of their activators, the Rho guanine nucleotide exchange factors (RhoGEFs). In this study, we provide the first characterization of the full-length protein encoded by RhoGEF TEM4 and delineate its domain structure, catalytic activity, and subcellular localization. First, we determined that TEM4 can stimulate guanine nucleotide exchange on RhoA and the related RhoB and RhoC isoforms. Second, we determined that TEM4, like other Dbl RhoGEFs, contains a functional pleckstrin homology (PH) domain immediately C-terminal to the catalytic Dbl homology (DH) domain. Third, using immunofluorescence analysis, we showed that TEM4 localizes to the actin cytoskeleton through sequences in the N-terminus of TEM4 independently of the DH/PH domains. Using site-directed mutagenesis and deletion analysis, we identified a minimal region between residues 81 and 135 that binds directly to F-actin and has an ∼90-fold higher affinity for ATP-loaded F-actin. Finally, we demonstrated that a single point mutation (R130D) within full-length TEM4 abolishes actin binding and localization of TEM4 to the actin cytoskeleton, as well as dampens the *in vivo* activity of TEM4 towards RhoC. Taken together, our data demonstrate that TEM4 contains a novel actin binding domain and binding to actin is essential for TEM4 subcellular localization and activity. The unique subcellular localization of TEM4 suggests a spatially-restricted activity and expands the diversity of mechanisms by which RhoGEF function can be regulated.

## Introduction

Rho family GTPases are molecular switches that cycle between inactive, GDP-bound and active, GTP-bound states [1]. RhoGEFs catalyze the exchange of bound GDP for GTP on Rho GTPases rendering them biologically active to signal to downstream effectors. The largest family of RhoGEFs in humans, with 68 human members, is the Dbl family of proteins [2], [3], which is characterized by a tandem catalytic Dbl homology (DH) and regulatory pleckstrin homology (PH) domain cassette responsible for accelerating the intrinsic nucleotide exchange activity of one or more members of the Rho family small GTPases. Together with DOCK family RhoGEFs, there are over 80 RhoGEFs that can act on 12 out of 20 Rho GTPases (8 Rho GTPases are predicted or verified to be constitutively activated and regulated by RhoGEF-independent mechanisms) [4]. This apparent signaling redundancy is perhaps most striking for activators of RhoA, where at least 24 RhoGEFs have been documented for this single Rho GTPase [2], [3]. Beyond the DH-PH cassette, Dbl family proteins are highly divergent and possess other interaction motifs or catalytic domains that account for the distinct mechanisms by which the activity of Dbl family RhoGEFs is regulated [2]. For example, the Dbl family RhoGEF, GEF-H1 is sequestered by microtubules through the C-terminal region of GEF-H1 and its release off the microtubular lattice promotes the spatial activation of RhoA and changes in the actin cytoskeleton essential for cellular migration [5]–[7]. Another Dbl family RhoGEF, Ect2 is sequestered in the nucleus during interphase and is released during mitosis to bind the centralspindlin complex to promote spatio-temporal activation of RhoA and formation of the actin-rich contractile ring that is essential for cytokinesis [8]–[10]. Thus, each RhoGEF can integrate distinct stimulus-dependent, spatio-temporally restricted Rho GTPase activation which leads to a rearrangement of the actin cytoskeleton essential for a variety of cellular processes.

In this report, we show that the RhoGEF, TEM4/ARHGEF17 [11], displays a subcellular localization unique amongst RhoGEF family members as it was associated with actin stress fibers. We found that TEM4 binds actin directly through a novel actin binding domain (ABD) within the N-terminus, and the TEM4 ABD is essential for TEM4 localization to the actin cytoskeleton and the *in vivo* activity.

## Materials and Methods

### Expression Constructs, Cell Culture and Reagents

A full-length TEM4/ARHGEF17 (NM_014786) 7.5 kb cDNA sequence was obtained by subcloning the exon (PCR amplified from human genomic DNA) encoding the first 456 N-terminal amino acids into the KIAA0337 clone (Kazusa DNA Research Institute, Japan) in pBS vector (Stratagene). cDNAs encoding full-length or truncation mutants of TEM4 were subsequently subcloned into the pEGFP mammalian expression vector (Clontech) and details are available upon request. Point mutations were introduced by site-directed mutagenesis. An expression vector encoding a fusion protein Lifeact-tRFP was made by subcloning cDNA sequences encoding the 17 amino acid actin binding peptide Lifeact [12] N-terminal to the red fluorescent protein TagRFP (Evrogen) into the pLL 5.0 lentiviral vector [13]. Antibodies: anti-GST (Sigma), anti-RhoC (Cell Signaling), anti-GFP (Clontech), anti-β-actin (Sigma), anti-α-tubulin (Sigma), and anti-TEM4 (ProSci).

Human umbilical vein endothelial cells (HUVECs; Clonetics) were maintained in EGM-2 supplemented with 10% fetal bovine serum (FBS; HyClone) and electroporated with expression constructs using Amaxa Nucleofection technology. NIH3T3 cells [14] were maintained in Dulbecco's Modified Eagle Medium (DMEM) supplemented with 10% calf serum and transfected using Lipofectamine Plus reagent (Invitrogen). 293T cells (ATCC) were maintained in DMEM supplemented with 10% FBS and transfected using the calcium phosphate DNA precipitation method. To visualize the actin cytoskeleton, HUVECs were transduced with lentivirus expressing tRFP-Lifeact.

**Figure 1:**
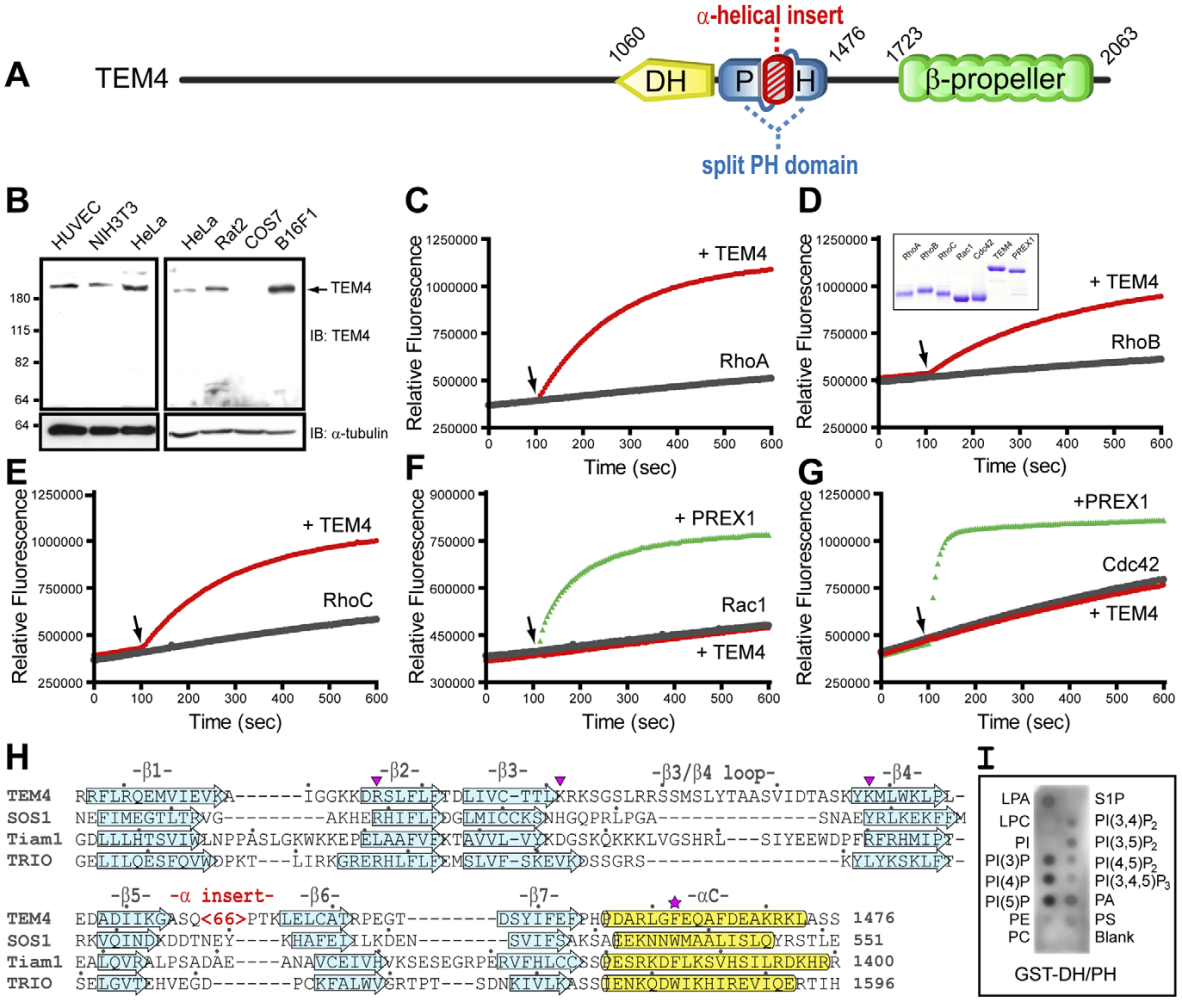
TEM4 is a Rho-specific guanine nucleotide exchange factor. A, A schematic representation of the domain structure of TEM4. *TEM4* encodes a 2063 amino acid protein that, in addition to the DH and PH domains, contains extensive N-terminal sequences (residues 1-1059) with no identifiable domains or motifs, and a C-terminal domain containing a predicted β-propeller fold (residues 1723–2039) likely consisting of seven WD40-related repeats [24], [60], [61]. The PH domain is split by an ∼60 amino acid α-helical insert. B, TEM4 is expressed in mammalian cell lines of endothelial and non-endothelial cell lineage. C–G, Guanine nucleotide exchange of RhoA (C), RhoB (D) or RhoC (E) (2 µM) was assessed in the presence (red trace) or absence (black trace) of the isolated DH-PH domain of TEM4 (10 nM). Guanine nucleotide exchange of Rac1 (F) and Cdc42 (G) (2 µM) was assessed in the presence of the DH-PH domains of TEM4 (50 nM; red trace) and PREX1 (200 nM; green trace). Intrinsic activities of Rac1 and Cdc42 are shown as a black trace. Arrows indicate time of GEF addition. Data shown are representative of three independent experiments. Five µg of each protein used in GEF assays were resolved by SDS-PAGE and visualized by Coomassie Blue dye (inset D). H, Structure-based sequence alignment of the TEM4 PH domain. A homology model of the PH domain of TEM4 (residues 1286–1476) was generated using the protein homology/analogy recognition engine^2^ (Phyre^2^) structure prediction server and then structurally aligned with the determined structures of DH-associated PH domains from SOS1 (PDB: 1AWE) [62], TIAM1 (PDB: 1FOE) [63] and TRIO (PDB: 1NTY) [64] using the Vector Alignment Search Tool (VAST) [65]. The assignments of beta strands (blue arrows, β1–β7) and the C-terminal α-helix (yellow cylinders, αC) are shown for each PH domain sequence. Secondary structure analysis predicts a ∼70 residue α-helical region (red) is inserted in the β5/β6 turn of the TEM4 PH domain. Residues within TEM4 likely utilized for phospholipid binding are marked (arrowheads). A highly conserved tryptophan residue within αC of PH domains (phenylalanine in TEM4 and Tiam1) is indicated (star). Dots mark every 10th residue. I, Protein-lipid overlay assay. Purified GST-DH-PH fusion protein was incubated with a lipid array and then detected with an anti-GST antibody. *LPA*, lysophosphatidic acid; *LPC*, lysophosphocholine; *PC*, phosphatidylcholine; *PS*, phosphatidylserine; *PA*, phosphatidic acid; *PE*, phosphatidylethanolamine; *S1P*, sphingosine 1-phosphate; *PI*, phosphatidylinositol; *PI(3)P*, phosphatidylinositol 3-phosphate; *PI(4)P*, phosphatidylinositol 4-phosphate; *PI(5)P*, phosphatidylinositol 5-phosphate; *PI(3,4)P_2_*, phosphatidylinositol 3,4-biphosphate; *PI(3,5)P_2_*, phosphatidylinositol 3,5-biphosphate; *PI(4,5)P_2_*, phosphatidylinositol 4,5-biphosphate; *PI(3,4,5)P_3_*, phosphatidylinositol 3,4,5-triphosphate.

### Fluorescent Microscopy

Twenty four h after electroporation, HUVECs plated in 35-mm glass bottom MatTek dishes were examined live using Zeiss LSM 510 or Zeiss Axio Observer spinning disk microscope with an oil immersion 63x NA 1.4 objective. NIH3T3 cells were transfected in 35-mm MatTek dishes and examined live using Zeiss LSM 510 within 24 h after transfection.

### Protein Purification and Guanine Nucleotide Exchange Assays

The cDNA fragments encoding residues 81–135 (ABD) or 1050–1478 (DH-PH) of TEM4 was subcloned into pProEx HTb (Invitrogen) in frame with an N-terminal glutathione S-transferase (GST) tag. TEM4 protein was expressed in Rosetta BL21 (DE3) *E. coli* cells (EMD Biosciences) for 5 h at 25°C, purified on a glutathione-coupled Sepharose column followed by an S200 size exclusion column (GE Healthcare), and concentrated in buffer A (20 mM Tris pH 8.0, 200 mM NaCl, 2 mM EDTA, 2 mM dithiothreitol and 10% glycerol). For some experiments the GST tag was subsequently removed by incubating GST-TEM4 with tobacco etch virus protease overnight and then passed over a glutathione Sepharose column equilibrated in buffer A to remove the cleaved GST.

To monitor TEM4 catalytic activity, N-methylanthraniloyl (mant)-GTP incorporation into bacterially-expressed and purified Rho GTPases was carried out with a FluoroMax-4 spectrometer (Horiba) at 22°C as described [15]. Two µM of relevant GTPases were loaded with 400 nM mant-GTP after TEM4 or P-Rex1 DH-PH domain proteins were added and the relative fluorescence (λ_em_  = 360 nm, λ_ex_  = 440 nm) was monitored.

**Figure 2:**
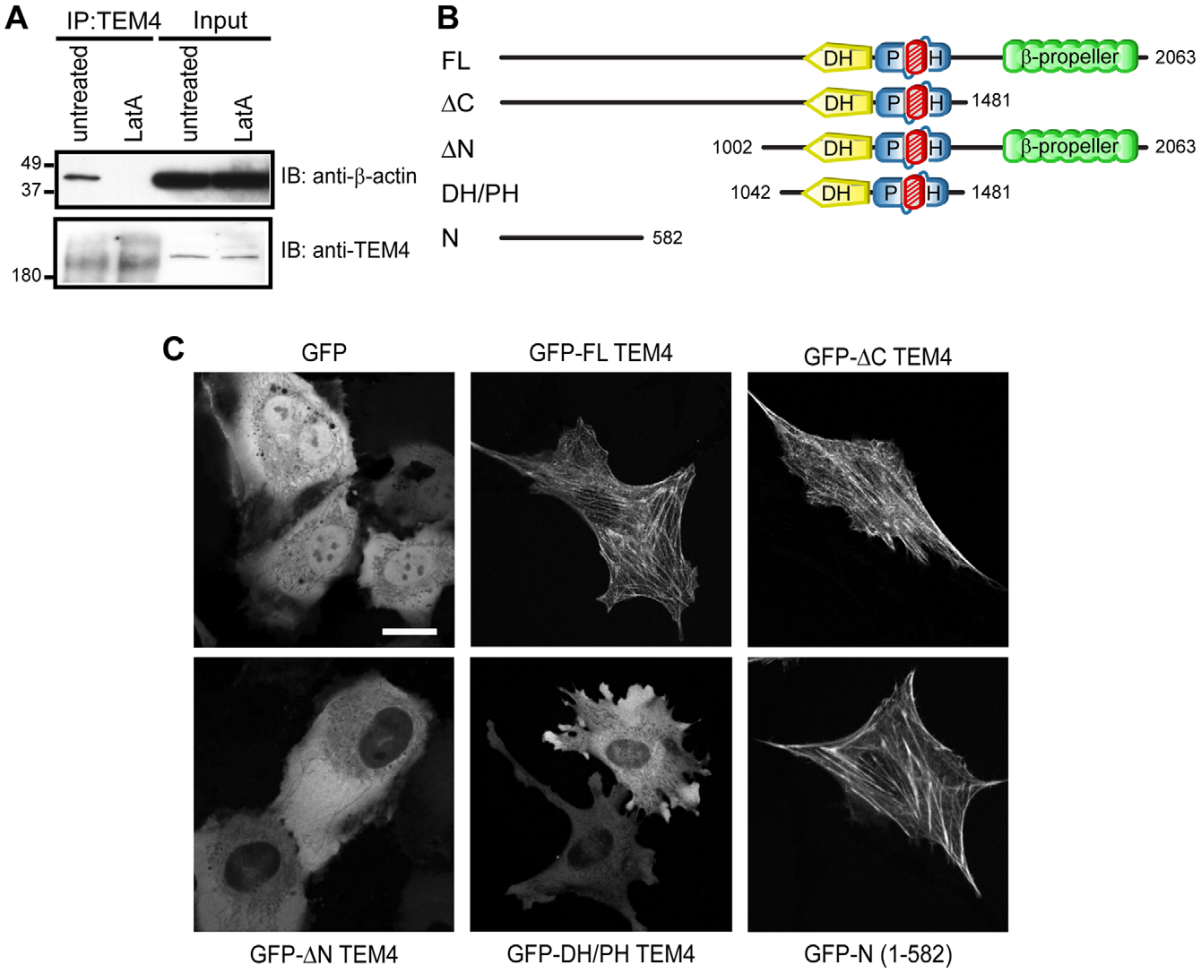
N-terminus of TEM4 is essential for localization of TEM4 to cytoskeleton. A, Endogenous TEM4 associates with F-actin. TEM4 was immunoprecipitated from HUVECs pretreated with LatA and β-actin was detected by western blotting. B, Schematic of GFP-TEM4 fusion constructs. *DH*, Dbl homology; *PH*, pleckstrin homology. C, The N-terminus of TEM4 possesses the actin association signal. HUVECs were electroporated with GFP or GFP-tagged TEM4 constructs as indicated and imaged live. Scale bar, 20 µm.

### Lipid Binding

To assess phosphoinositide binding to the TEM4 PH domain, PIP Strips (cat # P-6001; Echelon) were blocked with 3% fatty acid-free bovine serum albumin (Sigma) in phosphate-buffered saline, and incubated with 1 µg/ml recombinant GST-DH-PH for 1 h at room temperature. The strips were washed and immunoblotted with anti-GST antibodies.

### F-actin Co-sedimentation Assays

To evaluate TEM4 association with F-actin, high-speed actin co-sedimentation was performed as previously described [16], [17]. Briefly, G-actin (APHL99-A; Cytoskeleton) was diluted in G buffer (5 mM Tris-HCl pH 8, 0.1 mM CaCl_2_, 0.5 mM DTT, 0.2 mM ATP), converted to Mg-ATP-G-actin by incubating with 50 µM MgCl_2_, 0.2 mM EGTA and polymerized in the presence of 50 mM KCl, 1 mM MgCl_2_, and 1 mM ATP for 1 h at room temperature. GST-TEM4 was incubated with F-actin at room temperature for 30 min, and sedimented using an Airfuge (Beckman) at 23 psi (∼100,000×g) for 30 min. Supernatant and pellet fractions were solubilized in SDS sample buffer and resolved by SDS-PAGE. To calculate the binding affinity of TEM4 to F-actin, the supernatant-depletion method [17], [18] was used. The amount of TEM4 bound to F-actin was determined indirectly by immunoblotting the supernatant with anti-GST antibodies and quantified by densitometry. All blots were within the linear range of detection as determined by running out a range of GST-TEM4 protein dilutions (unpublished data). For experiments with ADP-G-actin, ATP-G-actin was converted to ADP-G-actin with 20 U/mL hexokinase (Sigma) and 1 mM glucose for 3 h at 4°C as previously described [16], [19] and used immediately.

**Figure 3:**
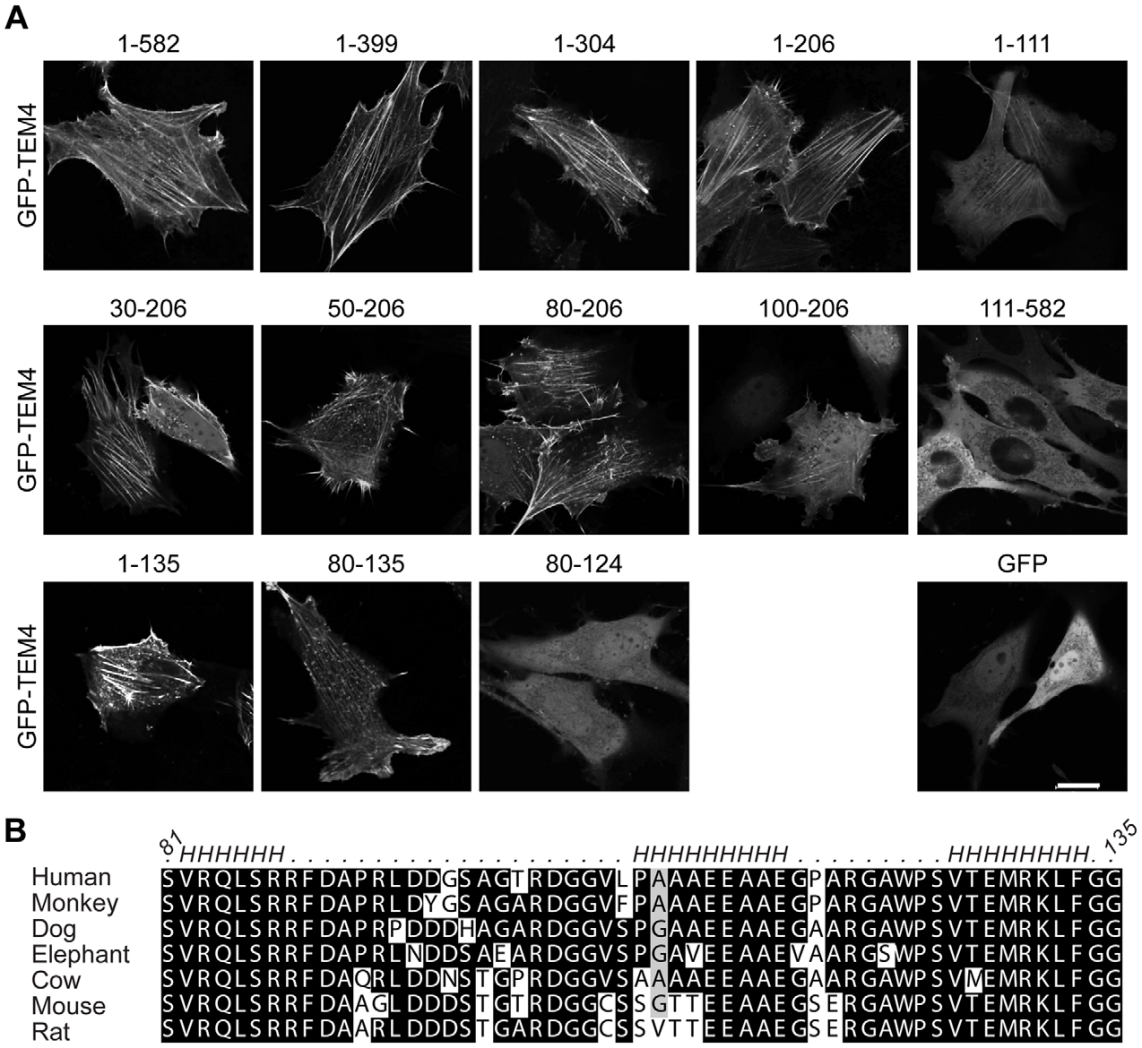
Detailed mapping of actin-association domain of TEM4 . A, GFP-TEM4 fusion constructs encoding various truncations of the N-terminus or GFP were transfected into NIH3T3 cells and imaged live. Scale, 20 µm. B, The newly identified actin-binding domain of TEM4 (aa 81–135 in human TEM4) is conserved among TEM4 orthologs. Helices (*H*) predicted by the secondary structure prediction server PredictProtein [66] are labeled.

### Immunoprecipitation Analysis

HUVEC were left untreated or preincubated with 1 µM LatA (Sigma) for 30 min and lysed in immunoprecipitation buffer (10 mM PIPES, pH 7, 100 mM KCl, 2 mM EDTA, 0.5% Triton X-100) containing protease inhibitors. Cell lysates were incubated with TEM4 antibody, precoupled to protein G Dynabeads (Invitrogen), for 4 h at 4°C. The beads were washed with the immunoprecipitation buffer four times and immunocomplexes were analyzed by immunoblotting.

### RhoC Activation Pull-down Assays

The pGEX construct encoding a GST fusion protein of the Rho binding domain (RBD) of Rhotekin (amino acids 7–89) (provided by Keith Burridge, UNC-Chapel Hill) was used for expression in bacteria and GST-Rhotekin-RBD was purified from bacterial cell lysates using glutathione-Sepharose 4B beads (Amersham Biosciences). Transiently transfected 293T cells were maintained in growth medium for 24 h and then serum-starved (0.5% FBS) overnight. Cells were lysed in lysis buffer (50 mM Tris, pH 7.5, 500 mM NaCl, 10 mM MgCl_2_, 1% Triton X-100, 10% glycerol) supplemented with protease inhibitors and pelleted at 13,000×*g* for 5 min. Thirty µg of GST-Rhotekin-RBD immobilized on glutathione-Sepharose 4B beads were incubated with 500 µg of clarified cell lysates for 1 h at 4°C. The beads were washed twice in wash buffer (25 mM Tris, pH 7.5, 40 mM NaCl, 30 mM MgCl_2_). Whole cell lysates and affinity-precipitated samples were subjected to SDS-PAGE and analyzed by western blotting using RhoC isoform-specific antibodies.

**Figure 4:**
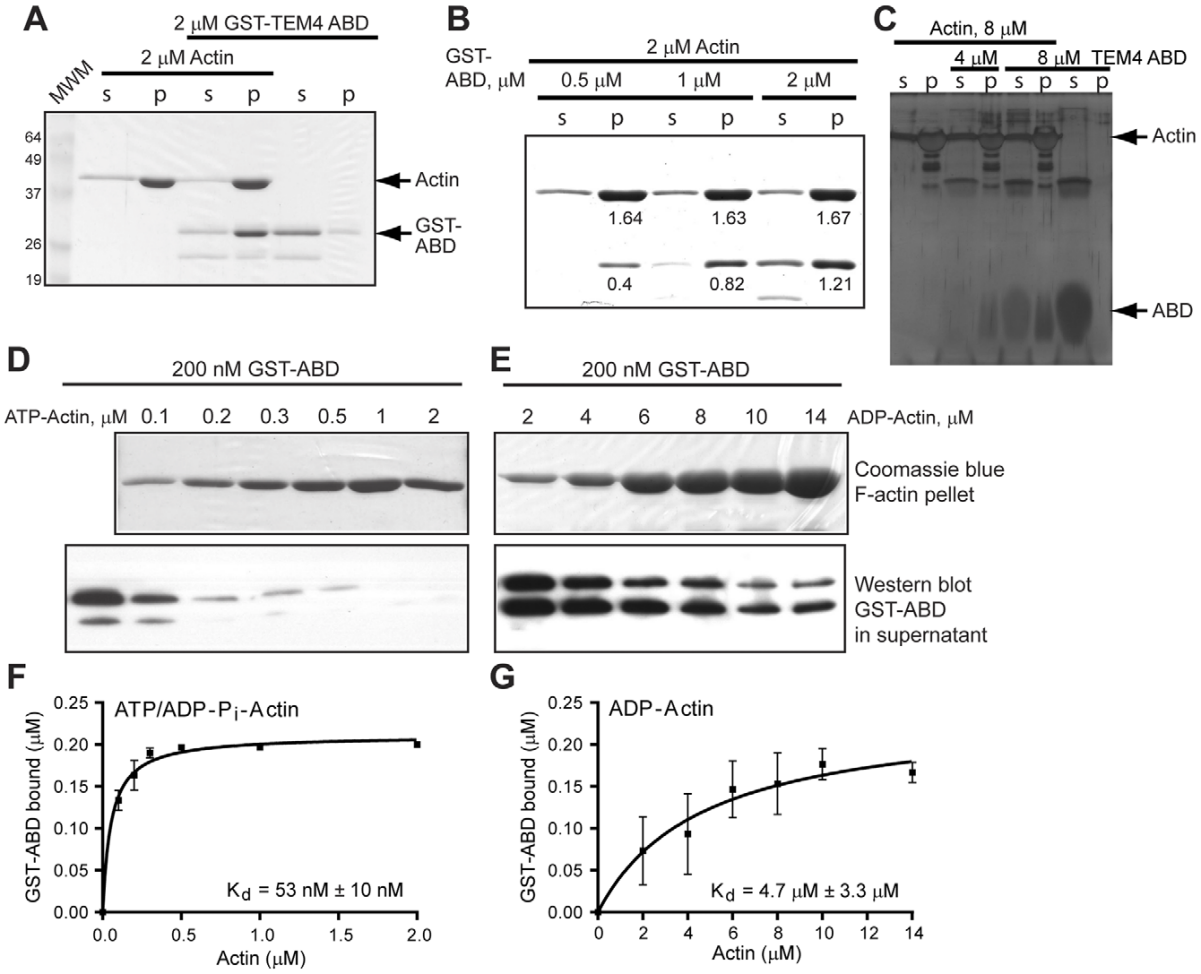
TEM4 binds directly to F-actin with high affinity . A, Binding of TEM4 (residues 81–135) to F-actin was examined by actin co-sedimentation assays. Recombinant GST-TEM4 protein was incubated with F-actin and subjected to high speed centrifugation. Soluble (s) and pellet (p) fractions were resolved by SDS-PAGE and stained with Coomassie Blue. B, Coomassie Blue-stained gel showing the near-saturation binding of GST-TEM4-ABD to F-actin. Numbers below protein bands indicate protein concentrations in µM as determined by densitometry. C, TEM4 ABD binds F-actin. Silver stained gel shows co-sedimentation of untagged TEM4 ABD with F-actin. D-G, TEM4 preferentially binds dynamic ATP/ADP-P_i_-F-actin. ATP- or ADP-bound G-actin was polymerized and binding of the resulting F-actin filaments to GST-ABD was determined by co-sedimentation analysis. Representative Coomassie Blue-stained gels showing amount of ATP/ADP-P_i_- or ADP-F-actin in the pellet of co-sedimentation experiment (D and E, top panel). Immunoblot showing amount of GST-ABD protein left in the supernatant in co-sedimentation experiments (bottom panel). F, G, Equilibrium binding of GST-ABD to ATP/ADP-P_i_- or ADP-F-actin. Amount of GST-ABD bound to F-actin was calculated from immunoblots similar to the ones shown in D and E.

## Results

### TEM4 is a Rho-specific RhoGEF

TEM4/ARHGEF17 is a member of the Dbl family of RhoGEFs [2] as it possesses a tandem Dbl homology (DH) and pleckstrin homology (PH) domain catalytic cassette. *TEM4* was annotated by St. Croix et al. [11] and encodes a 2063 amino acid protein with a predicted molecular weight of 222 kDa that, in addition to the DH and PH domains, contains extensive N-terminal sequences (residues 1–1064) with no predicted domains or motifs, and a C-terminal domain with a predicted seven-bladed β-propeller fold (residues 1469–2063) (Fig. 1A). Subsequently, another study reported on a 1510 amino acid protein encoded by *TEM4*, which they called p164-RhoGEF based on a predicted molecular weight of 164 kDa [20]. The p164-RhoGEF cDNA sequence was presumed to be full-length based on available EST clone data (KIAA0337). However, our additional EST sequence analyses determined that p164-RhoGEF is an N-terminal truncation of TEM4 sequence as originally annotated by St Croix et al., and this is now reflected in public DNA databases. We have further validated full-length TEM4 protein expression by western blot analysis with antibodies raised against epitope shared between TEM4 and p164-RhoGEF proteins. Consistent, with the current curated NCBI annotation, expression of only the full-length, p222 kDa TEM4 protein was detected in multiple human and rodent cell lines (Fig. 1B), confirming that the sequence of TEM4 used in this report constitutes the authentic full-length TEM4.

To determine the specificity profile of TEM4 towards Rho GTPases, a bacterially expressed DH-PH fragment of TEM4 (residues 1050–1478) was used in fluorescent-based GEF assays *in vitro* [15] using bacterially expressed recombinant Rho GTPases (Fig. 1C–G). In this assay, TEM4 accelerated the intrinsic guanine nucleotide exchange rate of RhoA, as previously shown [20], [21], and the closely related RhoB and RhoC isoforms (Fig. 1C–E), but not Rac1 or Cdc42 (Fig. 1F, G). The Rac1 and Cdc42 proteins were functional as shown by their ability to be stimulated by the isolated DH-PH domains of P-Rex1 [22]. These data demonstrate that the catalytic activity of TEM4 is specific for RhoA/B/C *in vitro*.

**Figure 5:**
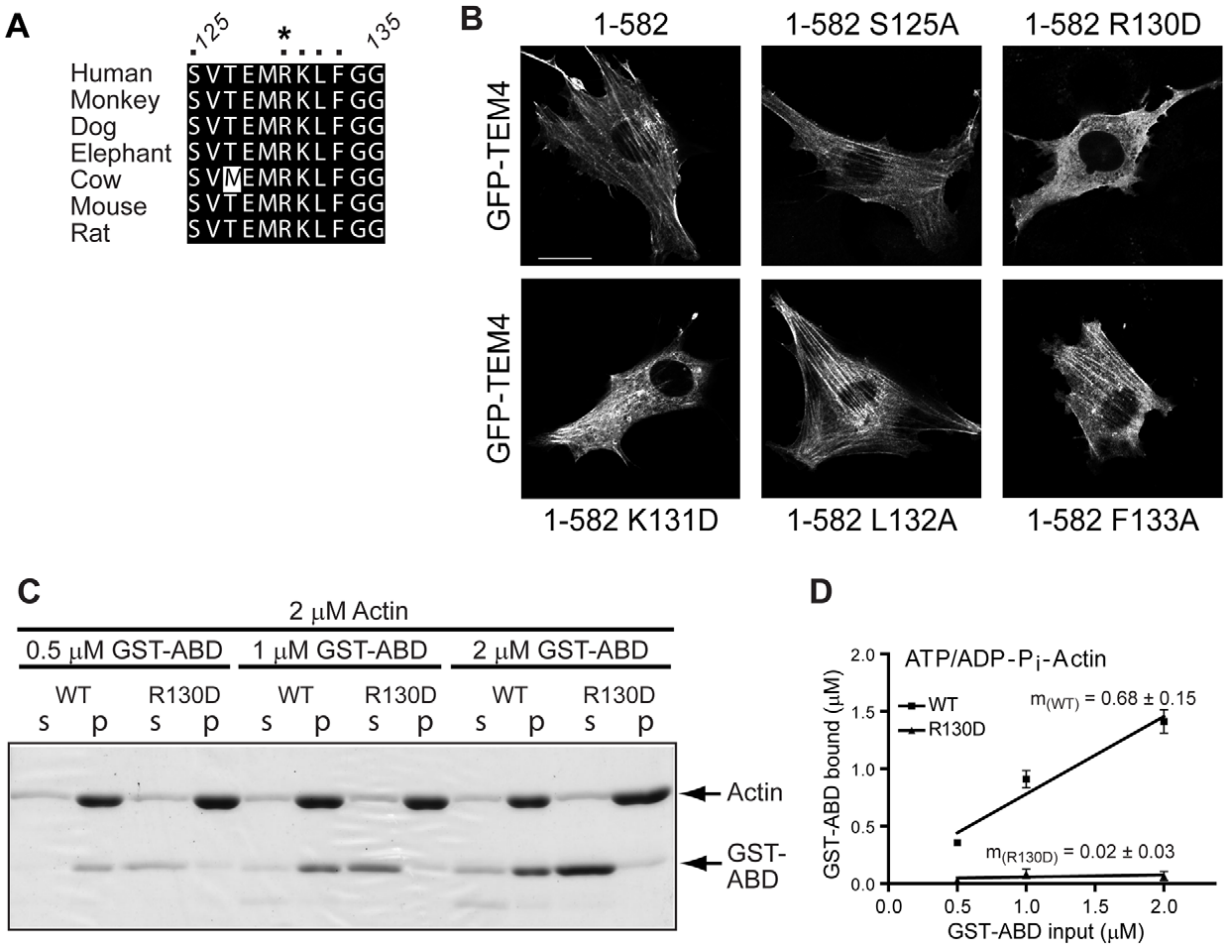
Arg 130 in TEM4 ABD is essential for F-actin binding. A, Alignment of residues 125-135 of human TEM4 with other species. Residues tested for actin binding *in vivo* are marked with a dot above the alignment. The arginine residue (R130), that we determined to be critical for binding to F-actin, is marked with the asterisk. B, Wild-type and point mutants of GFP-TEM4 1-582 were used to further map residues essential for actin association *in vivo* in live NIH3T3 cells. C, Coomassie Blue-stained gel comparing ATP/ADP-P_i_-F-actin binding capacity of wild-type (WT) and R130D mutant of GST-ABD in co-sedimentation experiments. D, Equilibrium binding of WT and R130D mutant of TEM4 ABD to ATP/ADP-P_i_-F-actin calculated from co-sedimentation experiments as shown in Fig. 4. Two independent co-sedimentation experiments were used to generate the data.

Finally, the presence of a PH domain C-terminal to the DH domain of TEM4 was put into question by a previous study [20]. Indeed, conventional domain prediction and annotation based solely on primary sequence alignment, such as is performed by the Simple Modular Architecture Research Tool (SMART) [23] does not identify a PH domain in TEM4 with statistical significance. However, three-dimensional structure prediction algorithms, which more reliably identify remote homologous sequences, readily detect a PH domain within TEM4 residues 1286–1476 (Fig. 1A, H). For example, the Phyre^2^ web server predicts a TEM4 PH domain with 99.4% confidence [24]. The Phyre^2^ generated PH domain homology model reveals that an ∼70 residue α-helical domain (aa 1366–1433) is inserted in the β5/β6 turn of the TEM4 PH domain. Furthermore, residues Arg 1306, Lys 1321 and Lys 1348 within the PH domain are likely to participate in forming a functional phospholipid binding site. To assess the ability of the PH domain of TEM4 to bind phosphoinositides [25], we performed lipid dot blot analyses using bacterially expressed GST-DH-PH fusion protein. We found that the TEM4 DH-PH domains bound primarily PI(3)P, PI(4)P and PI(5)P (Fig. 1I). Since DH domains do not characteristically bind phosphoinositides [26], [27], these results support the presence of a functional PH domain within TEM4.

**Figure 6:**
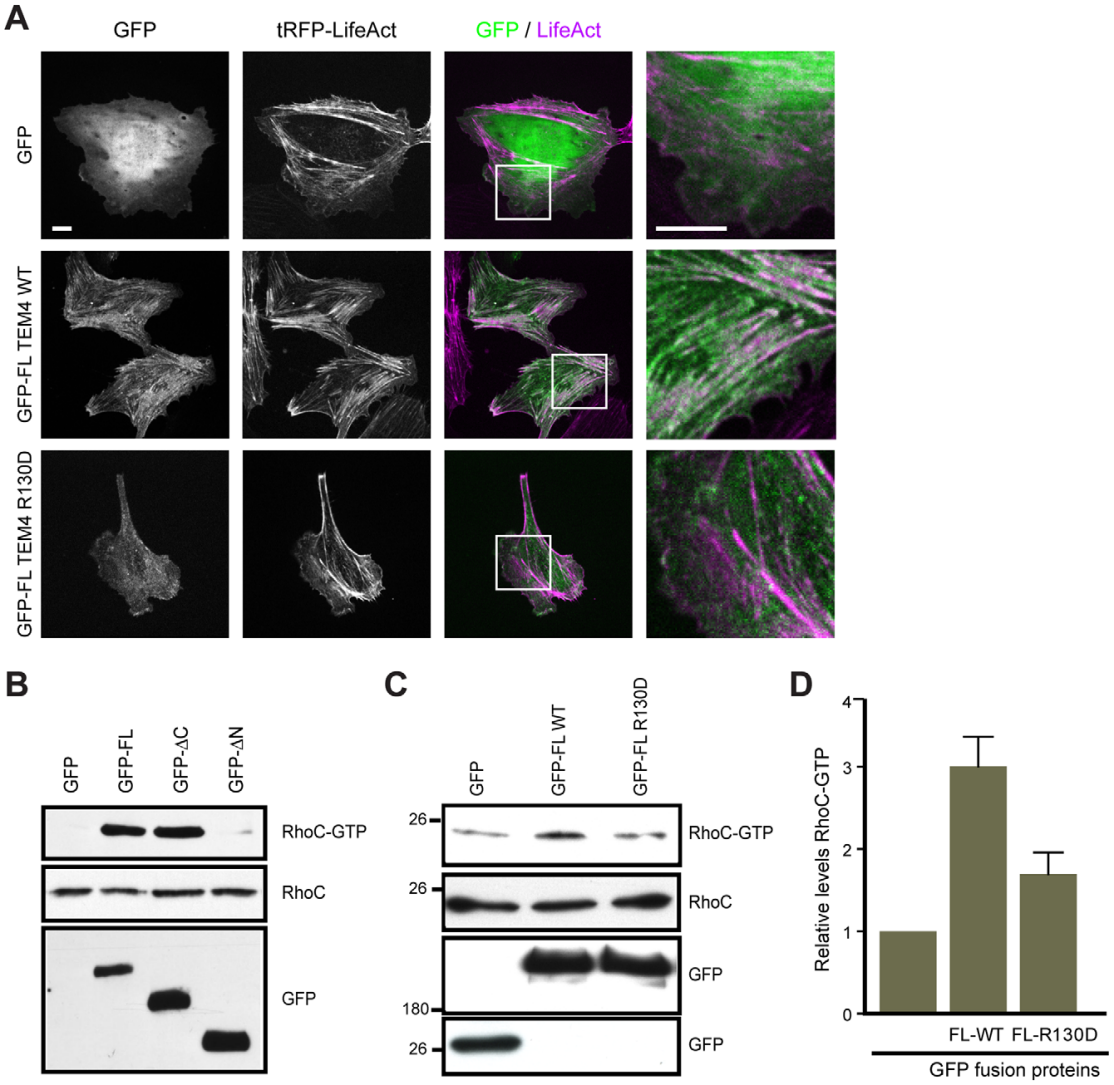
Actin binding is essential for subcellular localization and *in vivo* activity of TEM4. A, R130 is essential for the localization of TEM4 to actin stress fibers. HUVECs expressing GFP, GFP-FL TEM4 WT or R130D mutant were imaged live with tRFP-Lifeact marker. Scale bar, 10 µm. B, The N-terminus is essential for TEM4 *in vivo* activity. HUVECs expressing GFP-tagged TEM4 constructs were assessed for levels of active RhoC by affinity pull-down. C, Mutation of R130 impairs RhoC activation by TEM4. 293T cells were transfected with GFP-tagged wild-type or R130D mutant of TEM4 and levels of active RhoC were measured by affinity pull-down and presented in a bar graph (D). Data shown are representative of three independent experiments.

### The N-Terminus of TEM4 Regulates Subcellular Localization

Our initial studies aimed at determining interacting partners of TEM4 found actin to co-immunoprecipitate with exogenously expressed TEM4 (unpublished data). To confirm the TEM4-actin association, we immunoprecipitated endogenous TEM4 from primary human endothelial cells (HUVECs) that were left untreated or treated with an actin depolymerizing drug, latrunculin A (LatA) [28] prior to immunoprecipitation. As shown in Fig. 2A, endogenous TEM4 readily co-immunoprecipitated with actin, but the interaction was ablated in cells treated with LatA. Therefore, we concluded that TEM4 associates with F-actin filaments *in vivo*.

To investigate the association of TEM4 with F-actin further, the subcellular localization of full-length (FL) and truncated forms of TEM4 (Fig. 2B) was examined in live HUVECs. FL TEM4 (GFP-FL) resided on structures reminiscent of the actin cytoskeleton throughout the cytoplasm (Fig. 2C). HA-tagged FL TEM4 exhibited similar localization (unpublished data). Furthermore, while the deletion of the region C-terminal to the PH domain (GFP-ΔC) did not alter TEM4 localization, N-terminally deleted TEM4 (GFP-ΔN) and the isolated DH/PH domains (GFP-DH/PH) exhibited a diffuse cytoplasmic staining. These results suggest that sequences within the N-terminal portion of TEM4 are required for localization. Indeed, a construct encoding the first 582 N-terminal amino acids of TEM4 localized to the actin cytoskeleton (Fig. 2C). Therefore, sequences within the N-terminal region of TEM4 (residues 1–582) are necessary and sufficient for the subcellular localization of TEM4.

To identify the minimal domain required for the association with F-actin *in vivo*, we performed extensive deletion mutagenesis and identified a region encompassing residues 80–135 that exhibited cellular localization similar to TEM4 1–582 (Fig. 3A). The 81–135 region of human TEM4 is highly conserved within TEM4 orthologs (Fig. 3B), but does not exhibit sequence similarity to known actin-binding domains [29]–[31]. Therefore, TEM4 81–135 is sufficient for localization to the actin cytoskeleton *in vivo* and may constitute a novel actin binding domain (ABD).

### TEM4 Binds F-actin Through the Novel Actin-Binding Domain

To determine if the association of TEM4 with F-actin is direct, we purified recombinant GST-TEM4 81–135 (GST-ABD) and evaluated its ability to bind purified F-actin using a high-speed actin co-sedimentation assay. Recombinant GST-ABD was found predominantly in the F-actin-containing pellet (p) only in the presence of F-actin (Fig. 4A). The stoichiometry of GST-ABD binding to F-actin was estimated to be 1∶1.4 (Fig. 4B). However, when we accounted for the 15–20% GST cleavage product present in the GST-ABD protein sample, the stoichiometry came close to a 1∶1 ratio. The specificity of interaction between TEM4 ABD and F-actin was further confirmed by co-sedimentation of F-actin with the untagged TEM4 ABD (Fig. 4C). These data demonstrate that sequences within TEM4 81–135 comprise a high-affinity ABD.

In a cell, newly polymerized, dynamic actin filaments are composed mainly of ATP- and ADP-P_i_–actin [32], while mature filaments are composed primarily of ADP-actin [33], [34]. The nucleotide state of actin affects its interaction with actin binding proteins and affects kinetics of actin assembly and disassembly [17], [19], [34]. To determine if binding of TEM4 ABD to actin is dependent on the nucleotide state of actin, actin co-sedimentation assays were repeated using F-actin assembled from ATP- or ADP-G-actin. After ultracentrifugation, the amount of TEM4 bound to F-actin filaments was calculated from the amount depleted from the supernatant as detected by immunoblotting and densitometry (Fig. 4D, E). The data show that TEM4 ABD has a strong preference for binding dynamic F-actin, as it exhibited an approximately 90–fold higher affinity for filaments prepared from ATP-G-actin than those prepared from ADP-G-actin (Kd  = 53 nM and 4.7 µM, respectively; Fig. 4F, G). This affinity of the TEM4 ABD for ATP-F-actin is comparable to that measured for the actin nucleating complex, Arp2/3 which is 40 nM [35]. Taken together, these data demonstrate that TEM4 contains an ABD which binds preferentially to dynamic, newly assembled actin filaments.

Deletion of an 11 amino acid segment (residues 125–135) within the TEM4-ABD ablated the localization of TEM4 to the actin cytoskeleton *in vivo* (Fig. 3A). Therefore, it is highly probable that specific residues within 125–135 (Fig. 5A) contribute important interactions required for the high affinity binding of TEM4 to actin filaments. To identify individual residues essential for TEM4 binding to F-actin, we performed site-directed mutagenesis and determined the subcellular localization of these point mutants. Consistent with experimental data in other actin binding proteins where a positively charged surface was required for actin binding [36], mutation of the conserved R130 in TEM4 mislocalized GFP-TEM4 (Fig. 5B) and abolished the ability of GST-TEM4-ABD to co-sediment with ATP/ADP-P_i_ actin, suggesting that the R130 is critical for high affinity binding between TEM4 and F-actin (Fig. 5C, D).

### Arg 130 is Essential for TEM4 Subcellular Localization and In vivo Activity

We found that the N-terminus of TEM4 regulates its subcellular localization and R130 is essential for F-actin binding of the isolated ABD of TEM4 *in vitro*. To determine if actin binding is essential for the subcellular localization of FL TEM4 to the actin cytoskeleton, GFP-FL TEM4 WT or the R130D mutant were expressed in HUVECs together with the fluorescently-tagged (tRFP) actin marker, Lifeact, to visualize the actin cytoskeleton [12]. As shown in Fig. 6A, colocalization of GFP-FL TEM4 WT with the actin cytoskeleton was abolished by the introduction of the R130D mutation into TEM4. These data suggest that TEM4 contains a single ABD and that F-actin binding is essential for subcellular localization of TEM4 *in vivo*.

The subcellular localization of RhoGEFs may potentiate the activation of downstream Rho GTPases [2]. Therefore, we determined if the localization of TEM4 to the actin cytoskeleton modulates the *in vivo* activity of TEM4. We found that mislocalization of TEM4 to the cytosol by either amino-terminal deletion or mutation of R130 in the full-length protein severely reduced the ability of TEM4 to activate its substrate, RhoC, as compared to TEM4 WT (Fig. 6B–D). These data suggest that binding to F-actin, which is required for subcellular localization of TEM4, regulates TEM4 activity *in vivo*.

## Discussion

In this study, we characterized the RhoGEF, TEM4/ARHGEF17 and demonstrated that it exhibits a subcellular localization distinct among RhoGEFs facilitated by direct binding to F-actin through a novel actin binding domain. Furthermore, we found that disruption of TEM4 binding to actin caused an altered subcellular localization and impaired its *in vivo* activity. We suggest that this unique subcellular localization allows TEM4 to serve a highly distinct spatially-restricted role in regulating activation of Rho GTPases and therefore biological function.

RhoGEFs are large proteins distinguished by a unique set of protein and/or lipid interaction domains and motifs, outside of the shared DH-PH domains, that regulate function of RhoGEFs [2]. TEM4 possesses an actin binding domain (ABD) within the N-terminus (residues 81–135) that binds F-actin with high affinity. Our extensive analysis of the ABD using primary sequence analysis and secondary structure prediction algorithms failed to identify similarity to any known ABDs. A novel actin binding domain was previously reported in two other RhoGEFs, PDZ-RhoGEF and frabin [37], [38] and L/IIxxFE motif within these proteins was essential for binding to F-actin [39]. Interestingly, despite the lack of the overall sequence homology, TEM4 contains an LSxxFD motif within the N-terminus of the ABD; however, our studies suggest that the C-terminal residues are the most critical for TEM4 binding to F-actin. Regardless of the lack of primary sequence homology between the TEM4 ABD and known actin binding proteins, secondary structure prediction analysis suggests the presence of several helices (Fig. 3B) as common amongst ABDs [29]–[31], [40]. Finally, the ABD-containing region is absent from the two closely-related TEM4 family members, ARHGEF10 and ARHGEF10L/GrinchGEF [41], [42]. Therefore, we demonstrate that TEM4 contains a novel ABD and future studies are needed to characterize how TEM4 may affect actin filament dynamics.

Exogenous, GFP- or HA-tagged TEM4 readily decorated the entire length of actin stress fibers in HUVECs and endogenous TEM4 immunoprecipitates with F-actin. However, our *in vitro* actin-binding analysis found TEM4 ABD preferentially bound newly polymerized actin filaments, suggesting that TEM4 may associate with F-actin in the areas of the cell undergoing actin polymerization, *e.g.* leading edge, as shown for other actin-binding proteins with *in vitro* preference for ATP-F-actin [43]–[45]. Therefore, while the use of exogenously-expressed constructs was instrumental in mapping the ABD, the overexpressed protein may not accurately display the subcellular localization of endogenous TEM4. Thus, an important goal for our future studies will be an evaluation of the subcellular localization of endogenous TEM4.

Our data using affinity pull-down assays suggest that binding to actin regulates TEM4-induced activation of its Rho GTPase substrates. This could be achieved by at least two mechanisms. First, the ability of TEM4 to bind F-actin can result in recruitment and concentration of TEM4 at the sites of actin polymerization where Rho GTPases are known to be activated [46]. Second, like many other RhoGEFs [2], TEM4 may be auto-inhibited and ABD association with F-actin could relieve this autoinhibition, leading to enhanced intrinsic GEF activity similar to microtubular-mediated regulation of GEF-H1 [5]. Both of these scenarios would result in pools of active TEM4 localized in the areas undergoing actin polymerization where activation of Rho GTPases would be the most desirable to promote further cytoskeleton rearrangements.

Rho family GTPases are key regulators of actin cytoskeleton dynamics and proteins of Rho subfamily (RhoA, B, C) promote the formation of actin stress fibers [47]–[49] that are essential for a diversity of cellular processes, including cell shape, polarity, migration, cell-cell and cell-matrix interactions [47], [50], [51]. We have observed increased Rho GTPase activation and actin rearrangement in cells expressing WT and not the actin binding-deficient, R130D mutant of TEM4 (Fig. 6). These observations suggest that targeted localization of the catalytic activity of TEM4 may be critical for the subsequent actin rearrangement, which is essential for cellular functions of Rho GTPases such as cell migration and invasion [46], [52]–[54]. To our knowledge, this is the first report of the actin cytoskeleton-dependent regulation of an *in vivo* activity of a RhoGEF.

Rho GTPases interact with a diverse spectrum of downstream effectors and can regulate a diversity of cellular processes [55]. While RhoGEFs are best characterized for their abilities to facilitate Rho GTPase activation, there is also considerable evidence that RhoGEFs can influence the effector signaling output of their Rho GTPase substrates. One mechanism involves the restricted spatial activation of Rho GTPases [46], thereby leading to differential engagement and utilization of specific effectors. Another mechanism involves the function of RhoGEFs as a scaffold that can recruit effectors to promo­te differential effector utilization and signaling output [56], [57]. Interestingly, while the C-terminus of TEM4 was not required for subcellular localization, it is homologous to a region found in JSAP-1/JIP-3, a known scaffold for the c-Jun N-terminal kinase pathway [58], [59]. Future studies will determine if TEM4 promotes spatial activation of distinct effector pathways downstream of Rho GTPases and the mechanism of the preferential engagement of these pathways.

In conclusion, RhoGEF TEM4 binds F-actin through a novel actin binding domain and actin binding is essential for TEM4 subcellular localization and an *in vivo* activity. Future studies include regulation of TEM4-actin interaction and the biological consequences of actin-localized TEM4-Rho GTPase signaling.
